# Supplementary material for: Multiply fully recyclable carbon fibre reinforced heat-resistant covalent thermosetting advanced composites
Source: Nat Commun. 2017 Mar 2;8:14657. doi: 10.1038/ncomms14657 (PMC5337986; doi:10.1038/ncomms14657)
Supplement: Supplementary Information — Supplementary Figures, Supplementary Tables, Supplementary Methods, Supplementary Notes and Supplementary References [file ncomms14657-s1.pdf]

Supplementary Figures

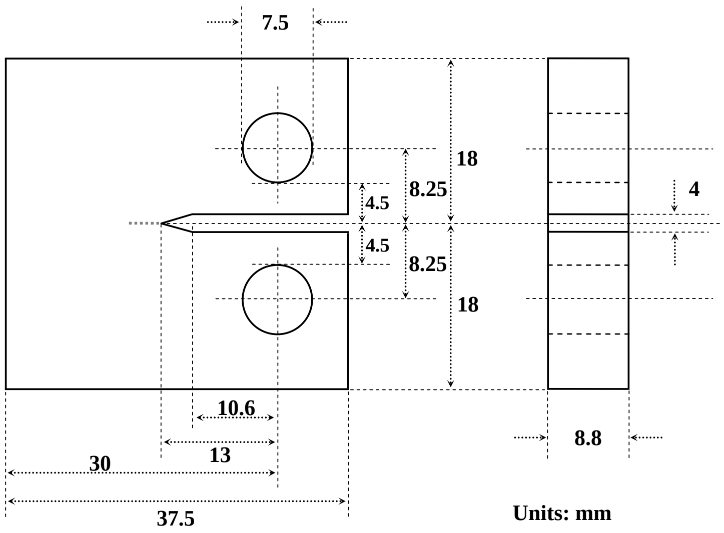

Supplementary Figure 1 | Configuration parameters of the CT specimen according to ASTM D5045-14 standard.

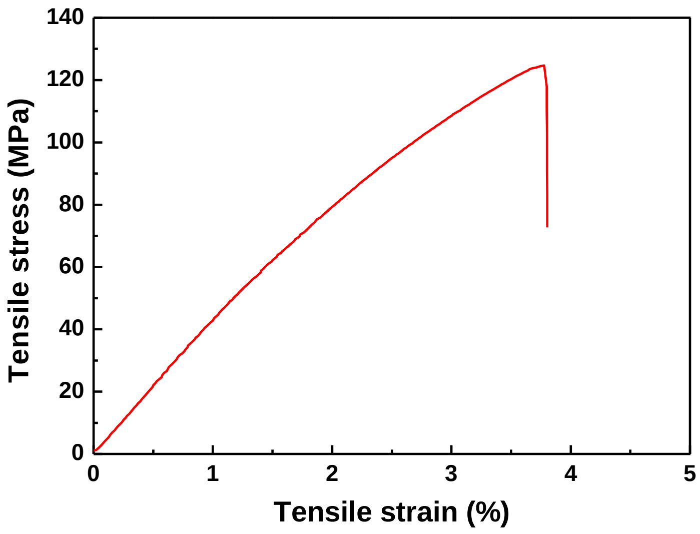

Supplementary Figure 2 | Representative tensile stress-strain curve of the BAPP-PHT.

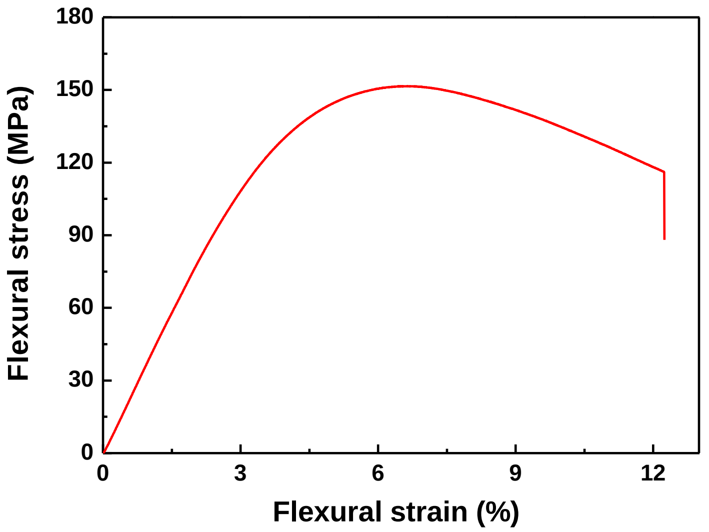

Supplementary Figure 3 | Representative flexural stress-strain curve of the BAPP-PHT.

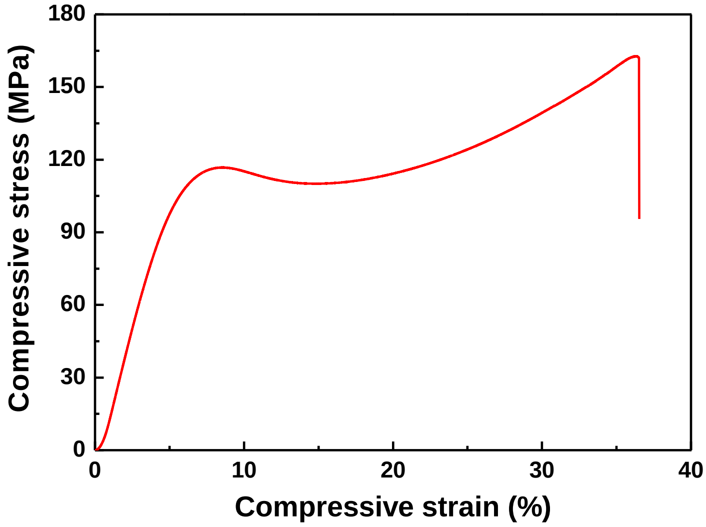

Supplementary Figure 4 | Representative compressive stress-strain curve of the BAPP-PHT.

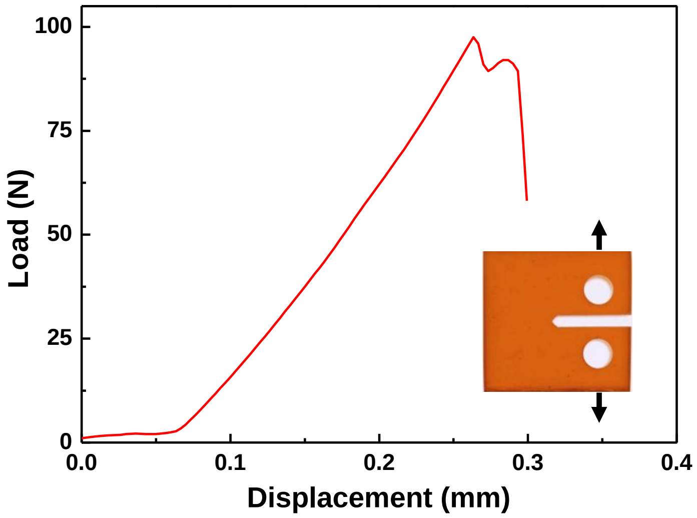

Supplementary Figure 5 | Representative load-displacement curve of the BAPP-PHT (The ratio of a/w is 0.501).

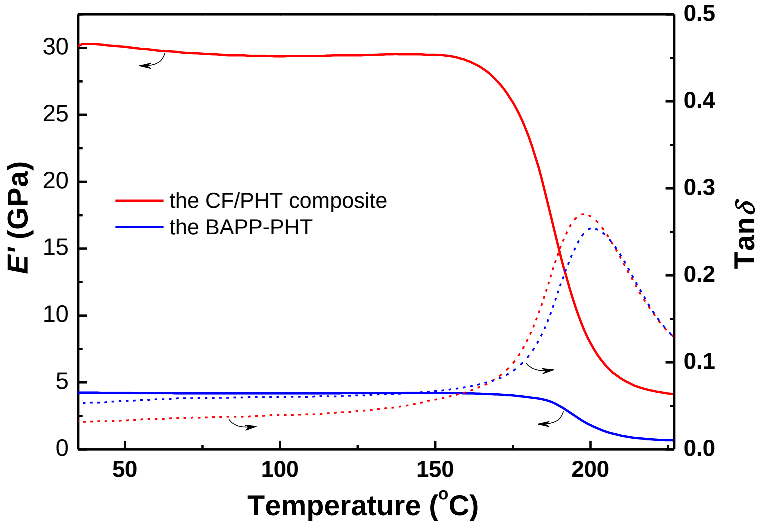

Supplementary Figure 6 | DMA results of the BAPP-PHT and the cross-ply CF/PHT composite.

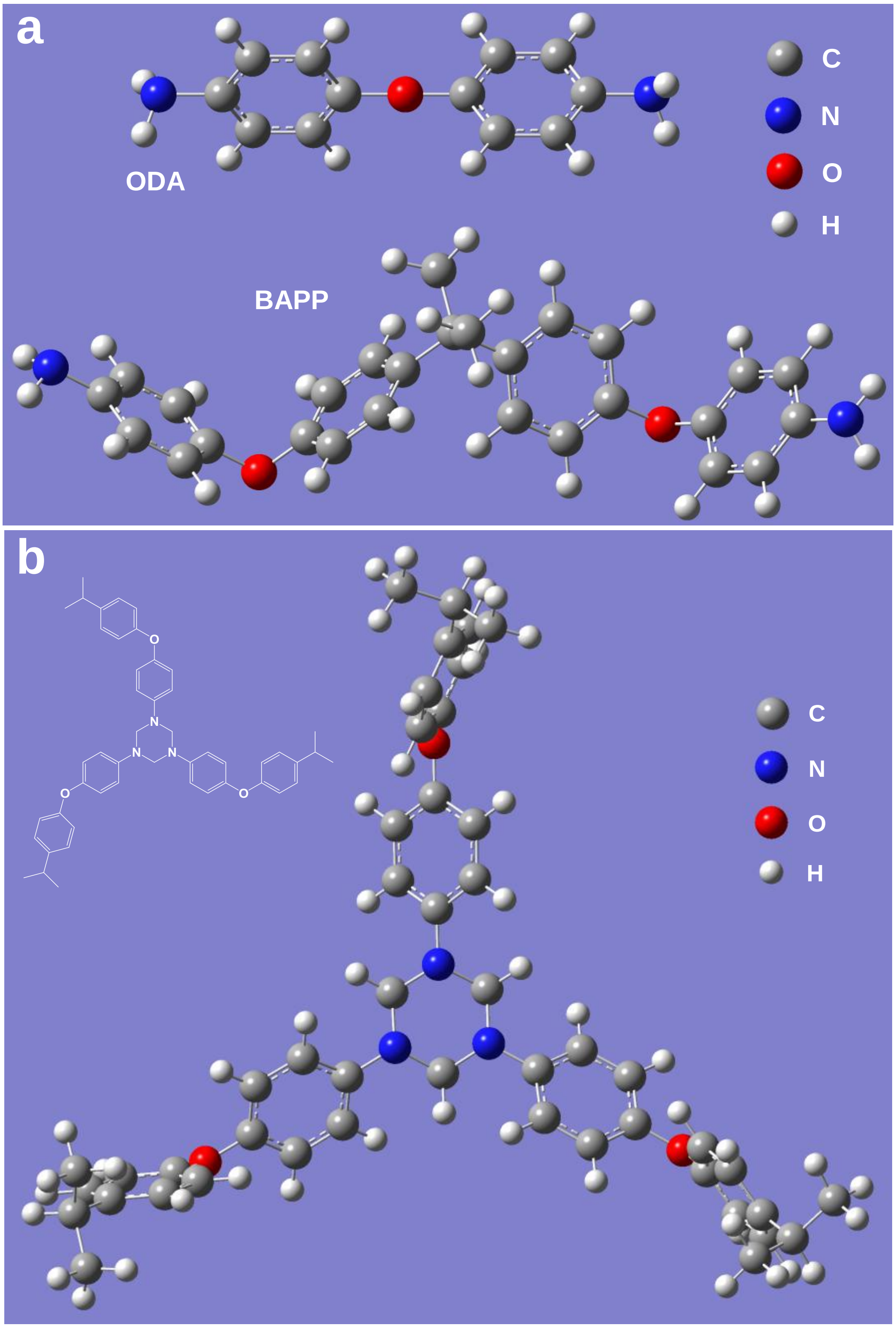

Supplementary Figure 7 | Molecular structure comparison between BAPP and ODA. (**a**) molecular structures of ODA and BAPP and (**b**) repeating structural unit of the BAPP-PHT. All quantum calculations were implemented with the Gaussian 09 software package[1]; The initial geometries were manually constructed by GaussView 5.0 graphical interface[2]; The ground-state structural optimizations for all molecular models were carried out without imposing any symmetric constraints by adopting the dispersion-corrected B3LYP density functional theory (DFT) with the 6-31G(d) basis set[3-5]; The concomitant vibrational frequency calculations were used to verify that each optimized structure lies in a true minimum point on the potential energy surface.

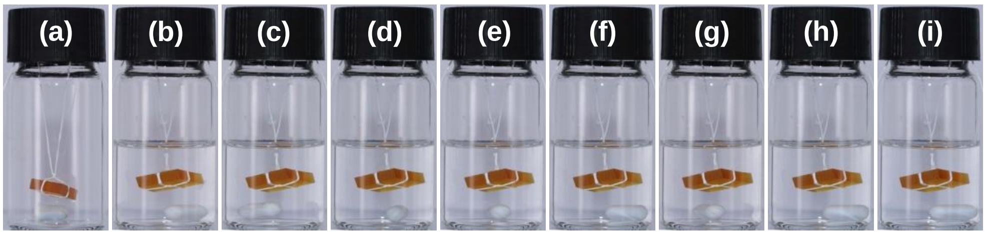

Supplementary Figure 8 | The resistance evaluation of the BAPP-PHT to chemical reagents. (**a**) Resin blank, (**b**) 0 h after the addition of distilled water. The test times of (**c**)~(**i**) are (**c**) 1 day, (**d**) 2 days, (**e**) 3 days, (**f**) 4 days, (**g**) 5 days, (**h**) 6 days and (**i**) 7 days, respectively.

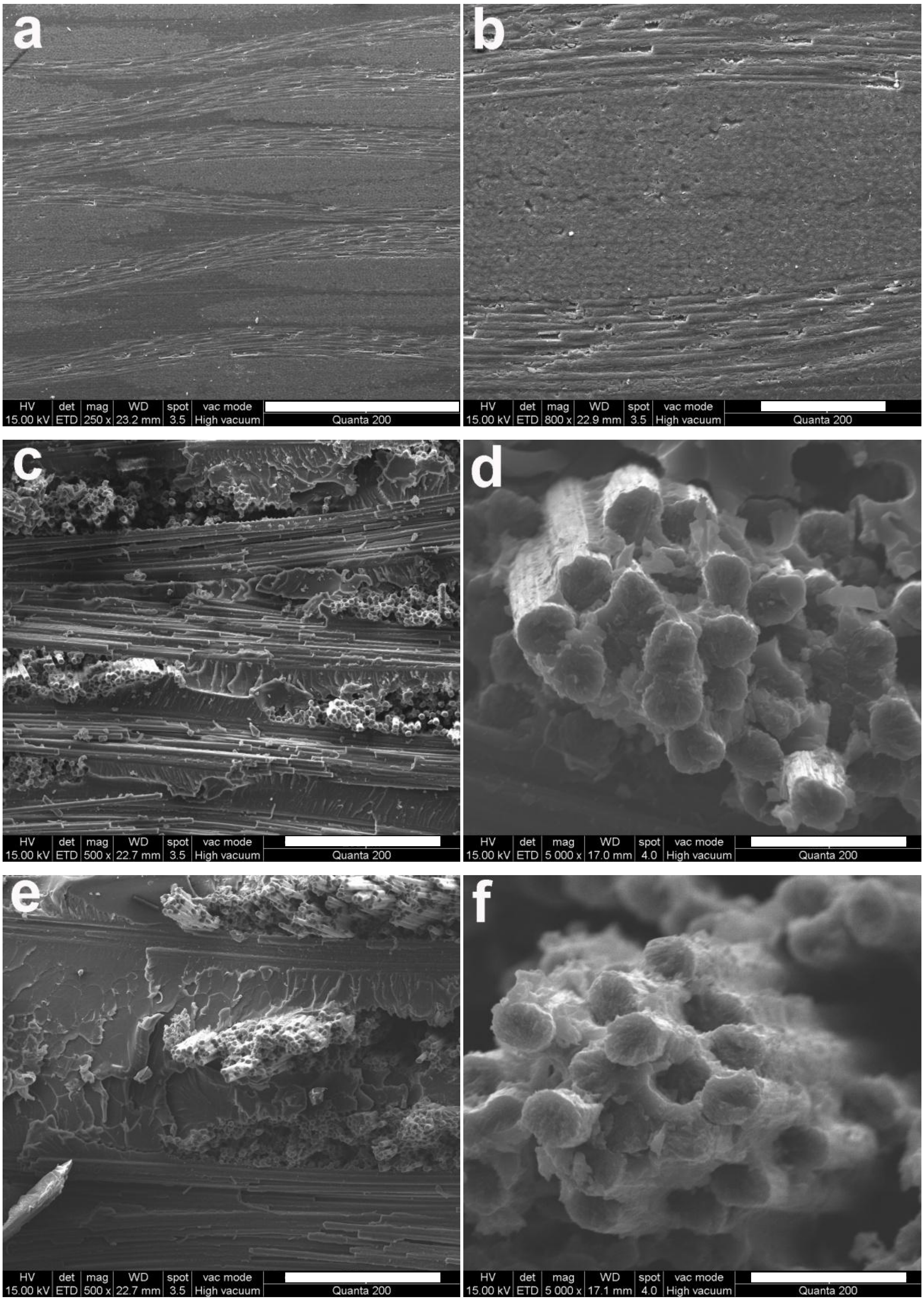

Supplementary Figure 9 | Structure characterization of the cross-ply CF/PHT composites. (**a**) and (**b**) SEM images of the cross section of the cross-ply laminate. (**c**) and (**d**) SEM images of the fracture planes of the cross-ply composites for tensile test. (**e**) and (**f**) SEM images of the fracture planes of the cross-ply composites for flexural test. Scale bars: (a), 500 μm; (b), 100 μm; (c), 200 μm; (d), 20 μm; (e), 200 μm; (f), 20 μm.

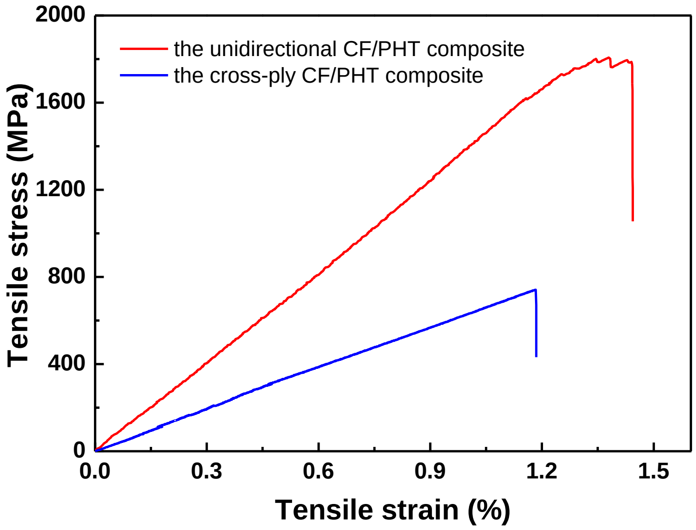

Supplementary Figure 10 | Representative tensile stress-strain curves of the cross-ply CF/PHT composite and unidirectional CF/PHT composite. The test result of the unidirectional composite comes from the 0° direction samples.

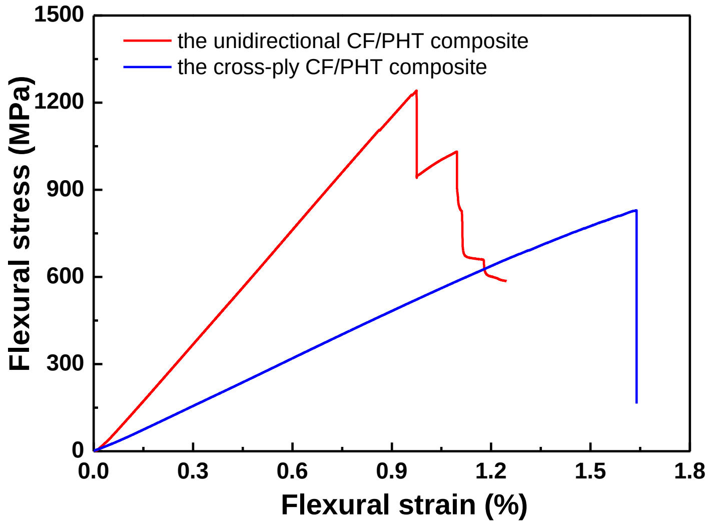

Supplementary Figure 11 | Representative flexural stress-strain curves of the cross-ply CF/PHT composite and unidirectional CF/PHT composite. The test result of the unidirectional composite comes from the 0° direction samples.

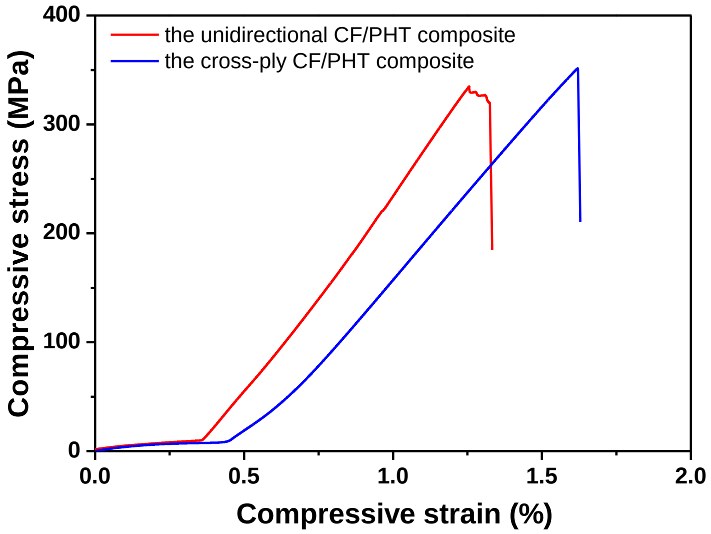

Supplementary Figure 12 | Representative compressive stress-strain curves of the cross-ply CF/PHT composite and unidirectional CF/PHT composite. The test and calculation are carried out according to the compression test method of thin composite laminates described in reference 37. The test result of the unidirectional composite comes from the 0° direction samples.

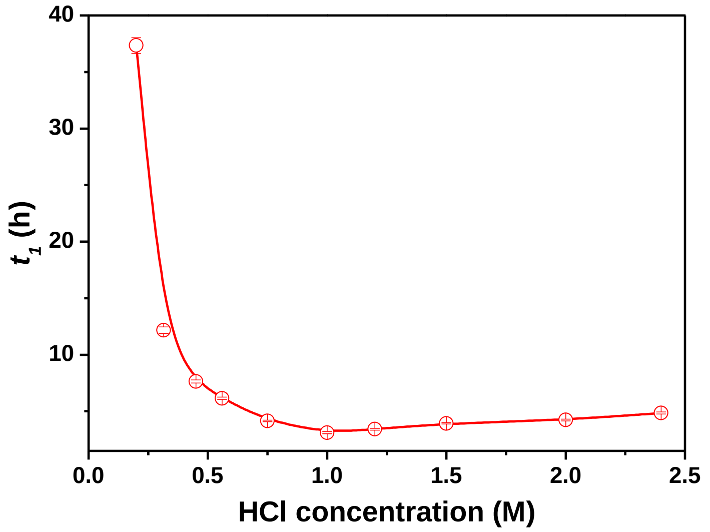

Supplementary Figure 13 | The influence of HCl concentration on the first-stage depolymerization time (t_1) of the BAPP-PHT with the same THF ratio (H_2O/THF=2/8).

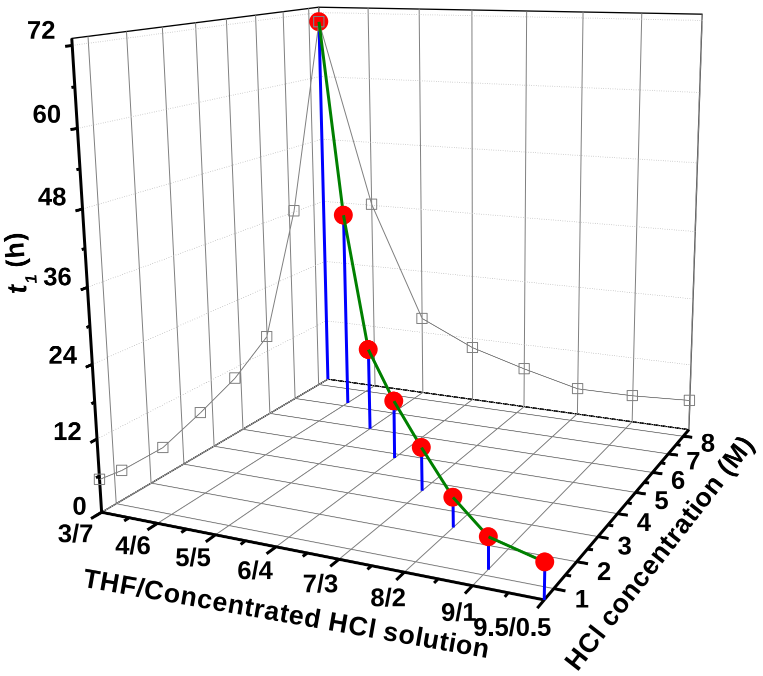

Supplementary Figure 14 | The influenceof HCl concentration and THF ratio on the first-stage depolymerization time (t_1) of the BAPP-PHT.

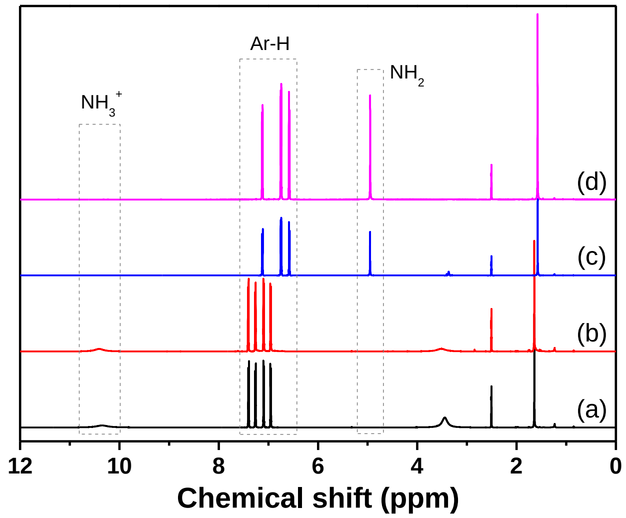

Supplementary Figure 15 | ^{1}H NMR spectra of the control samples and the gray white precipitate produced at 18 h of the BAPP-PHT degradation process in 1M HCl/THF mixed solution at room temperature. (**a**) the BAPP hydrochloride prepared by dissolving BAPP into THF, adding excess 1 M HCl aqueous solution to precipitate and further filtering and drying. (**b**) the gray white precipitate obtained by filtering, washing with THF and drying. (**c**) the recovered product from the precipitate by successively cleaning with saturated sodium bicarbonate solution, THF and distilled water and drying. (**d**) the BAPP raw material.

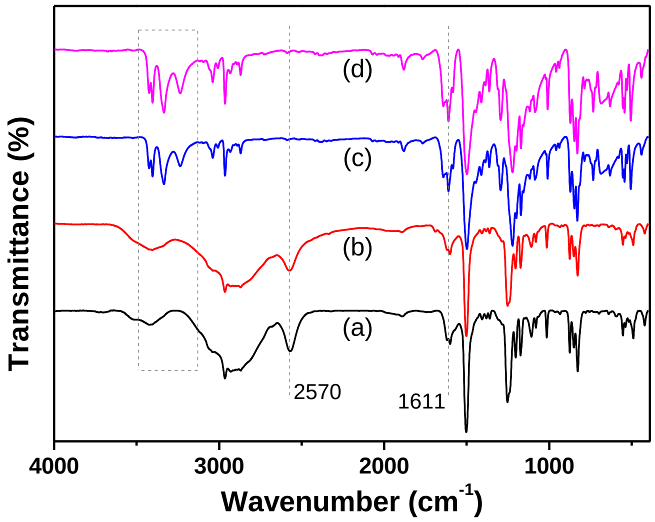

Supplementary Figure 16 | FTIR spectra of the control samples and the gray white precipitate produced in at 18 h of the BAPP-PHT degradation process in 1 M HCl/THF mixed solution at room temperature. (**a**) the BAPP hydrochloride prepared by dissolving BAPP into THF, adding excess 1 M HCl aqueous solution to precipitate and further filtering and drying. (**b**) the gray white precipitate obtained by filtering, washing with THF and drying. (**c**) the recovered product from the precipitate by successively cleaning with saturated sodium bicarbonate solution, THF and distilled water and drying. (**d**) the BAPP raw material.

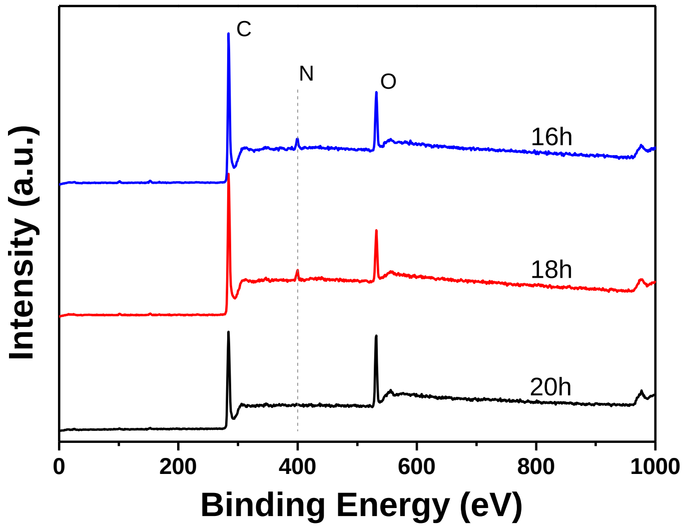

Supplementary Figure 17 | XPS spectra of the CF cloths recyced at different depolymerization times of the cross-ply CF/PHT composites.

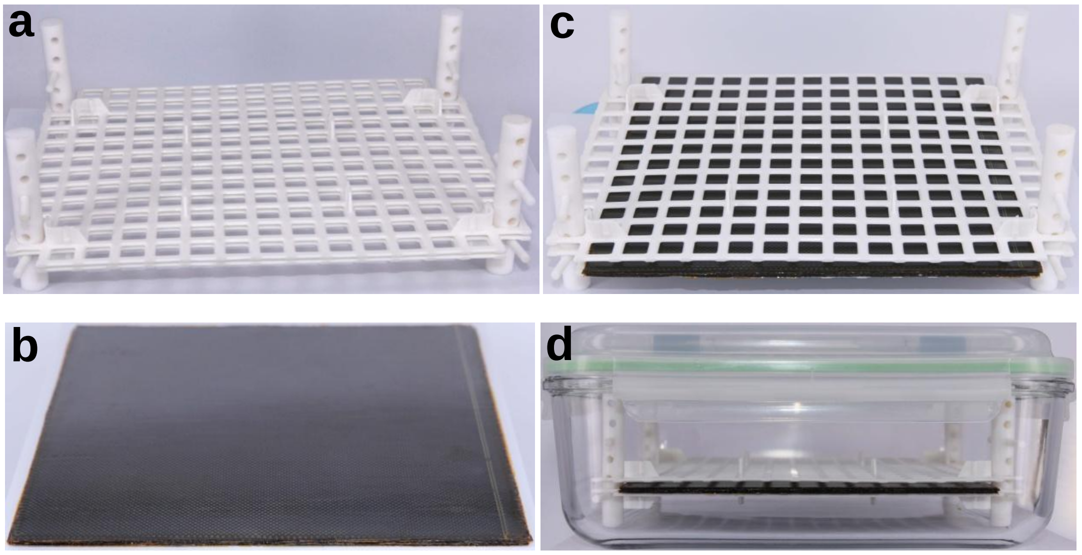

Supplementary Figure 18 | The degradation device used for the CF/PHT composite laminate with a size of 20 cm×20 cm×3 mm (L×W×H). (**a**) the PTFE fixture with freely adjustable grid space. (**b**) the cross-ply composite laminate speciman prepared with 24 layers 0.124 mm thick plain weave CF cloth prepregs. (**c**) the fixture with the cross-ply composite laminate specimen. (**d**) the sealable glass vessel used to settle the fixture.

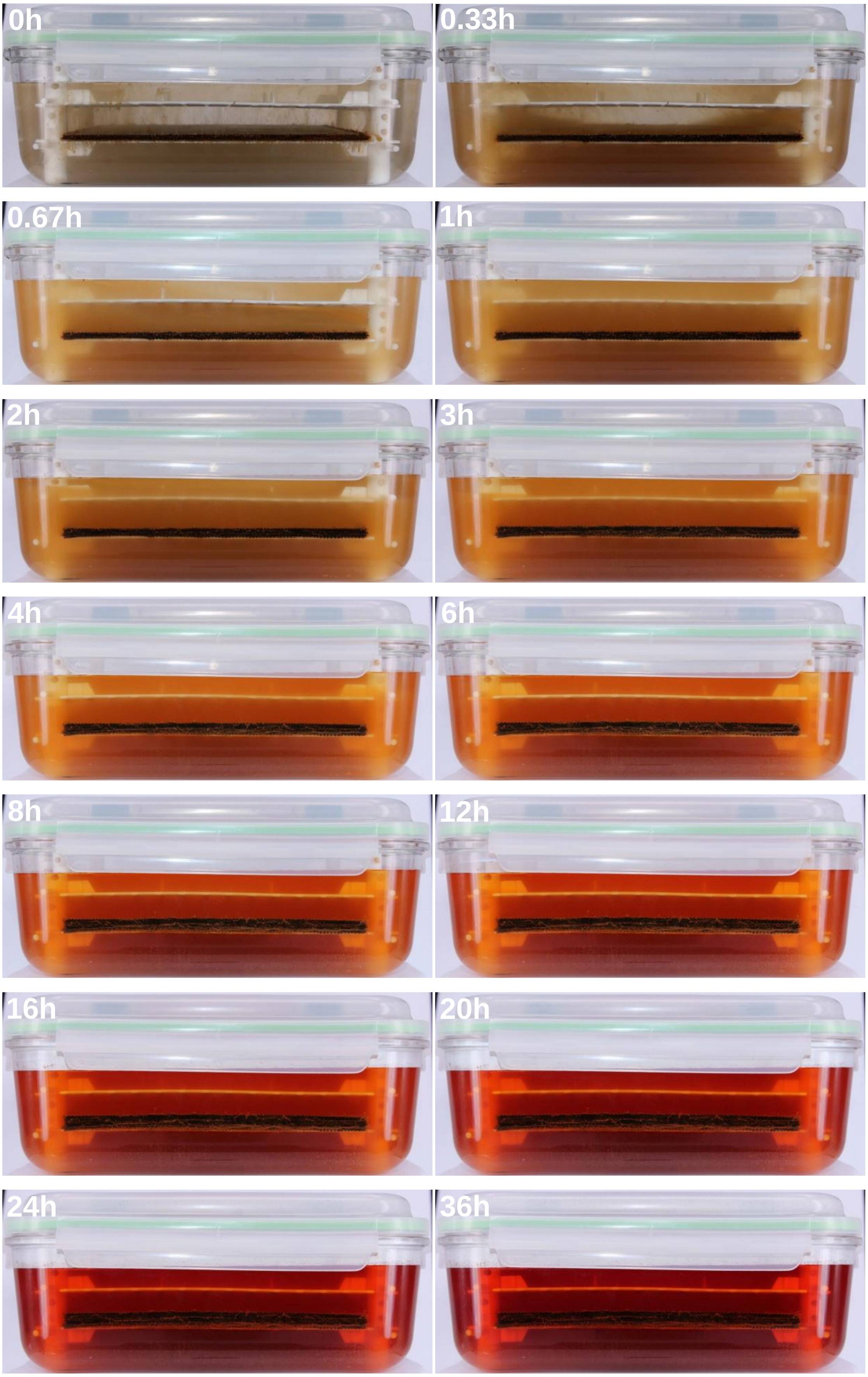

Supplementary Figure 19 | The degradation process of the cross-ply CF/PHT composite laminate in 3L 1 M HCl/THF mixed solution at different times.

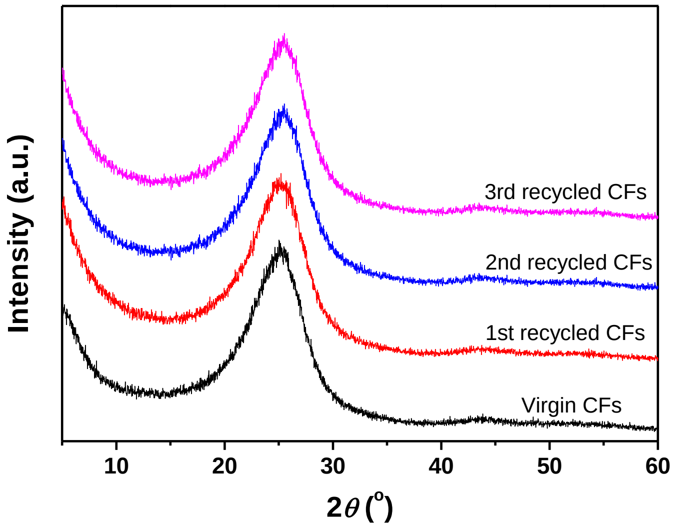

Supplementary Figure 20 | XRD spectra of the virgin CFs and the recycled CFs of different recycles.

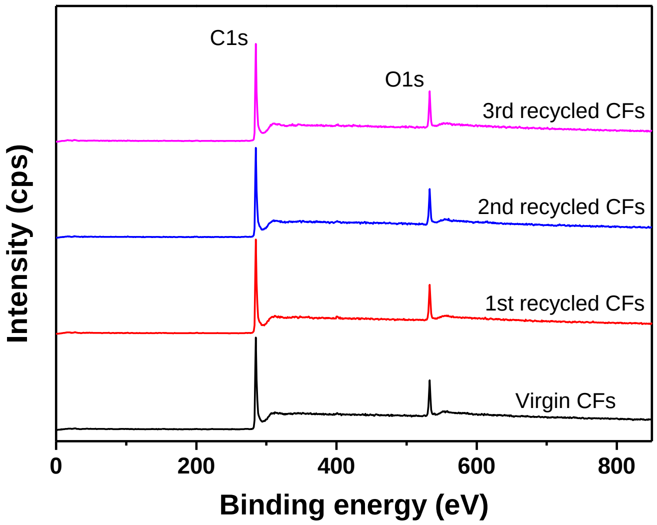

Supplementary Figure 21 | XPS survey spectra of the virgin CFs and the recycled CFs of different recycles.

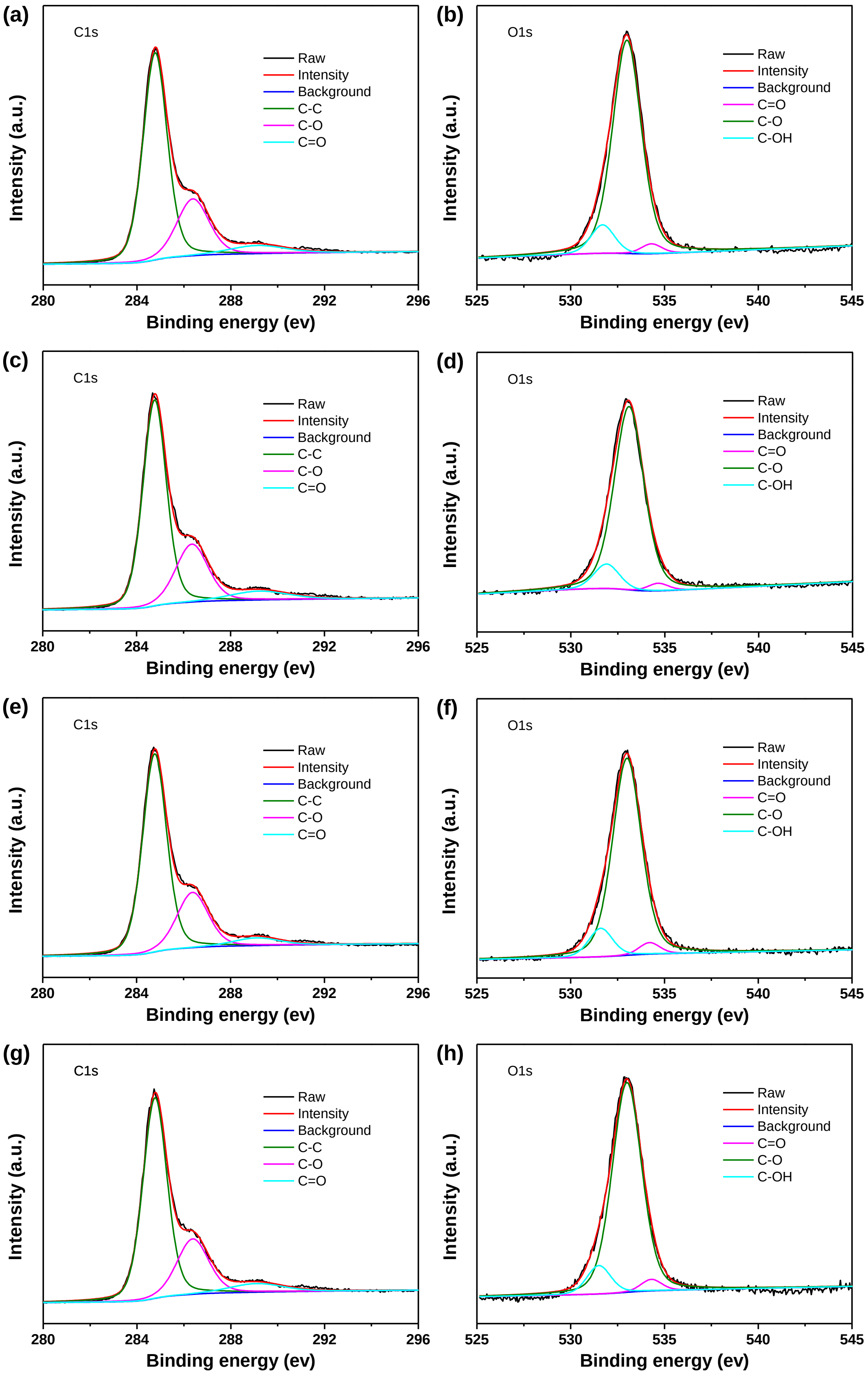

Supplementary Figure 22 | XPS spectra of the virgin and recycled CFs. (**a**) and (**b**) the virgin CFs. (**c**) and (**d**) the 1st recycled CFs. (**e**) and (**f**) the 2nd recycled CFs. (**g**) and (**h**) the 3rd recycled CFs.

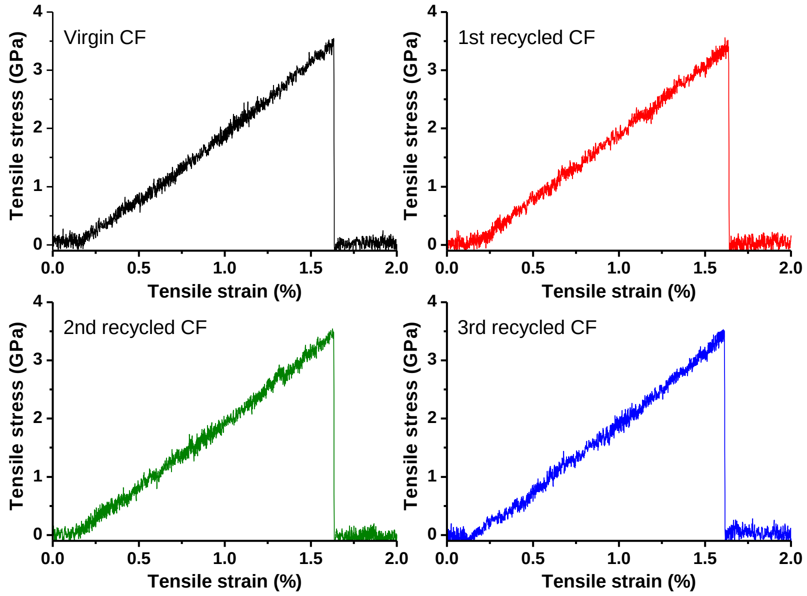

Supplementary Figure 23 | Representative monofilament tensile stress-strain curves of the virgin CFs and the recycled CFs of different recycles.

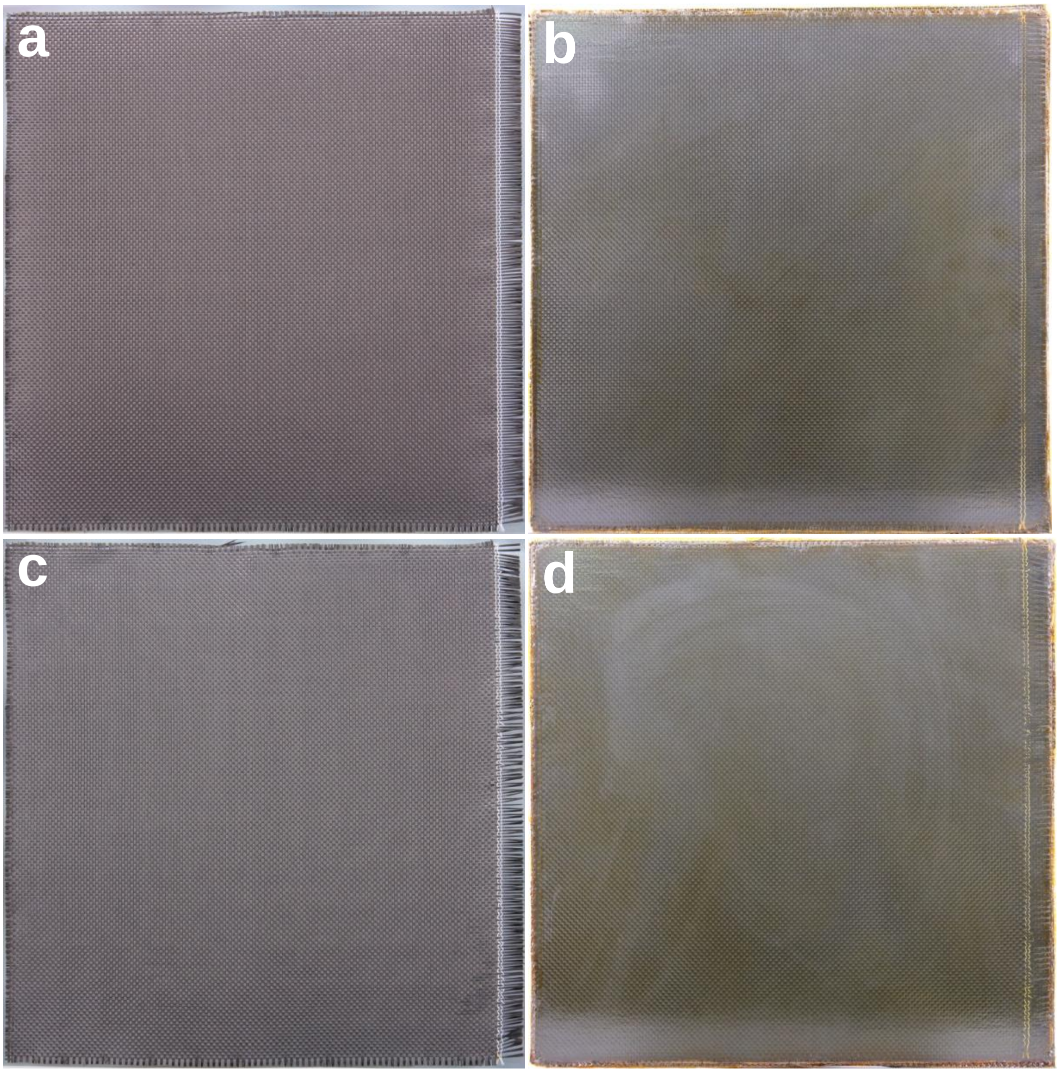

Supplementary Figure 24 | Photos of the virgin and recycled/regenerated CF cloths and cross-ply composite laminates. (**a**) The marked virgin CF cloth. (**b**) The composite laminate prepared with (**a**) as the outermost layer. (**c**) The once-recycled CF cloth of (**b**) in 3.6 M HCl/THF (H_2O/THF=3/7) mixed solutions for 48 h at room temperature. (**d**) The regenerated composite laminate prepared with (**c**) as the outermost layer. The white marks on the surface of the composite laminates are caused by the residual release agents and light interference during photographing.

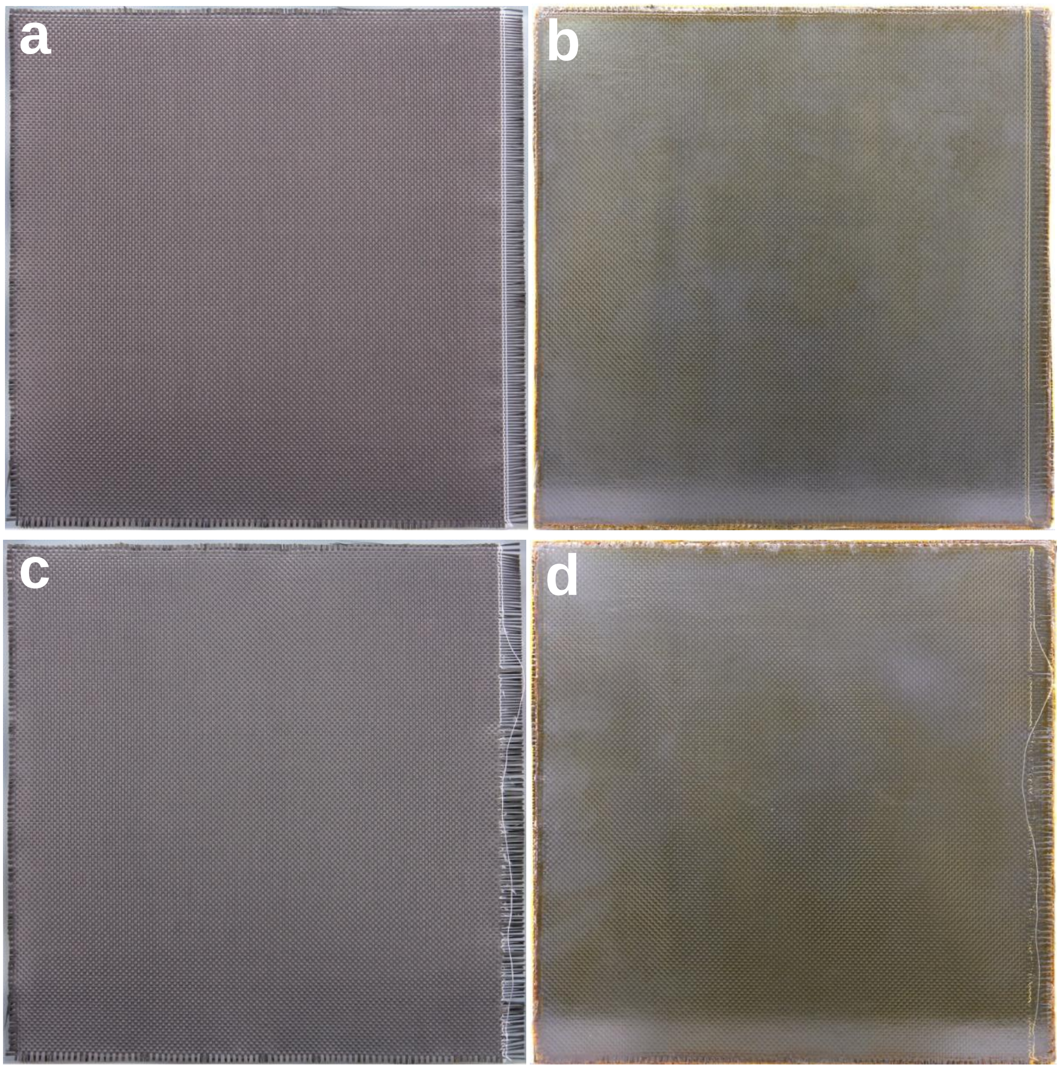

Supplementary Figure 25 | Photos of the virgin and recycled/regenerated CF cloths and cross-ply composite laminates. (**a**) The marked virgin CF cloth. (**b**) The composite laminate prepared with (**a**) as the outermost layer. (**c**) The once-recycled CF cloth of (**b**) in 4.8 M HCl/THF (H_2O/THF=4/6) mixed solutions for 60 h at room temperature. (**d**) The regenerated composite laminate prepared with (**c**) as the outermost layer. The white marks on the surface of the composite laminates are caused by the residual release agents and light interference during photographing.

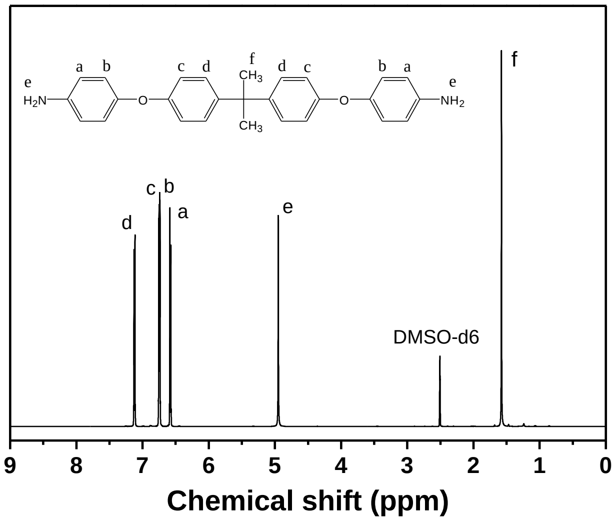

Supplementary Figure 26 | ^{1}H NMR spectrum of BAPP in DMSO-d6.

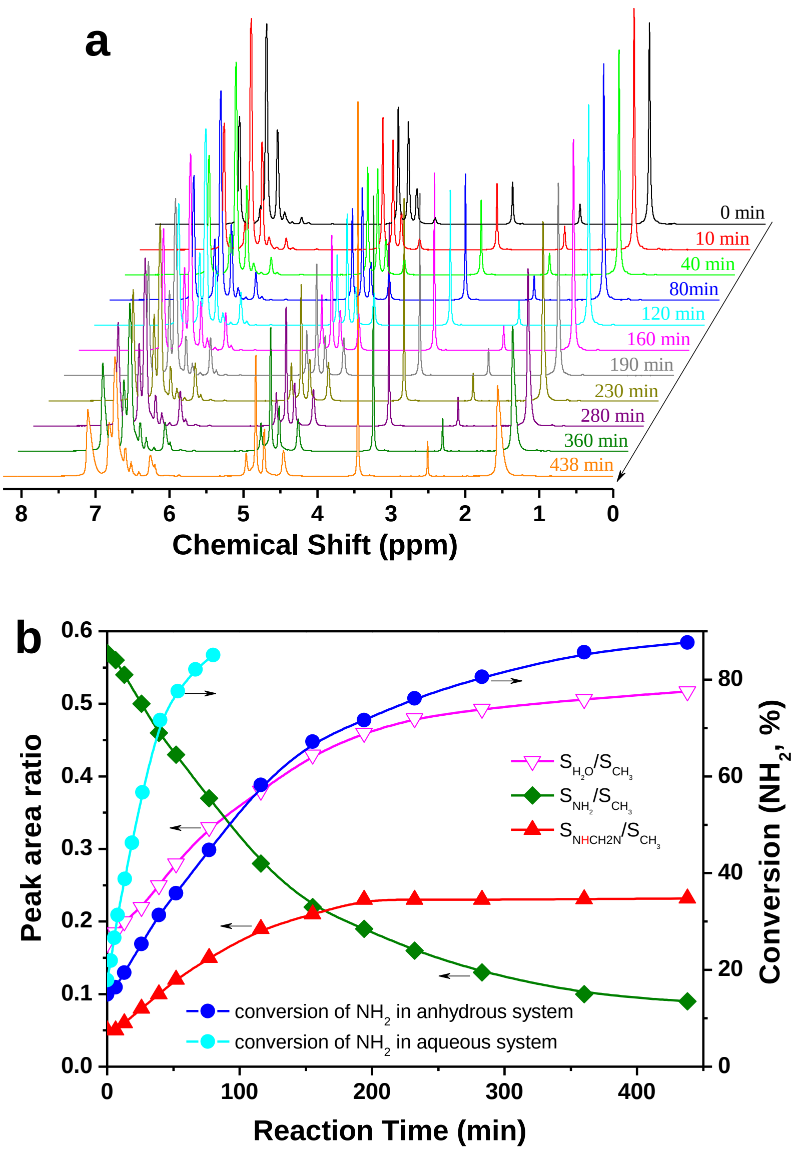

Supplementary Figure 27 | The polymerization kinetic curves of BAPP and formaldehyde in anhydrous DMSO-d6. (**a**) ^{1}H NMR spectra of the reaction system at different times. (**b**) the peak area ratios between diferent groups during the reaction process and the conversion kinetic curves of amino group under different systems. Methyl proton peak area at near 1.55 ppm is used as internal standard. The conversion data of amino group in aqueous system (10vol% H_2O) comes from Supplementary Fig. 28.

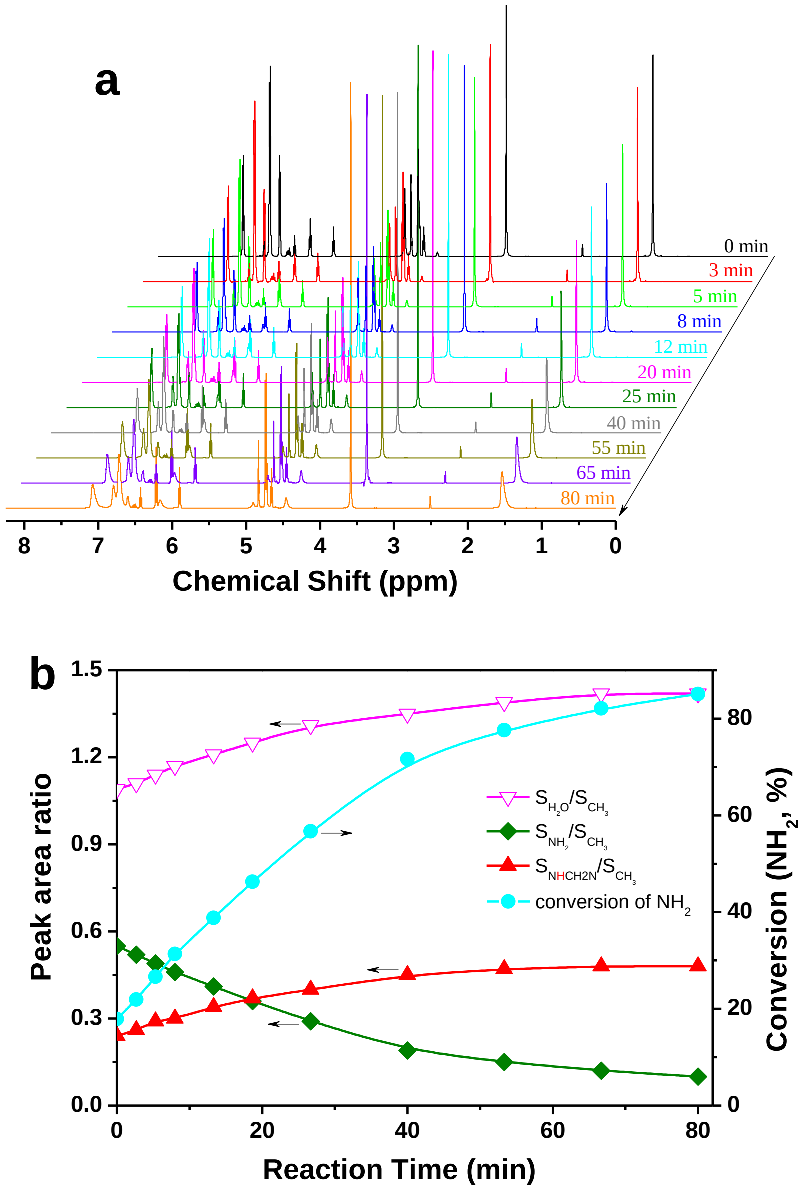

Supplementary Figure 28 | The polymerization kinetic curves of BAPP and formaldehyde in aqueous DMSO-d6 (containing 10vol% H_2O) reaction system. (**a**) ^{1}H NMR spectra of the reaction system at different times. (**b**) the peak area ratios between diferent groups during the reaction process and the conversion kinetic curve of amino group. Methyl proton peak area at near 1.55 ppm is used as internal standard.

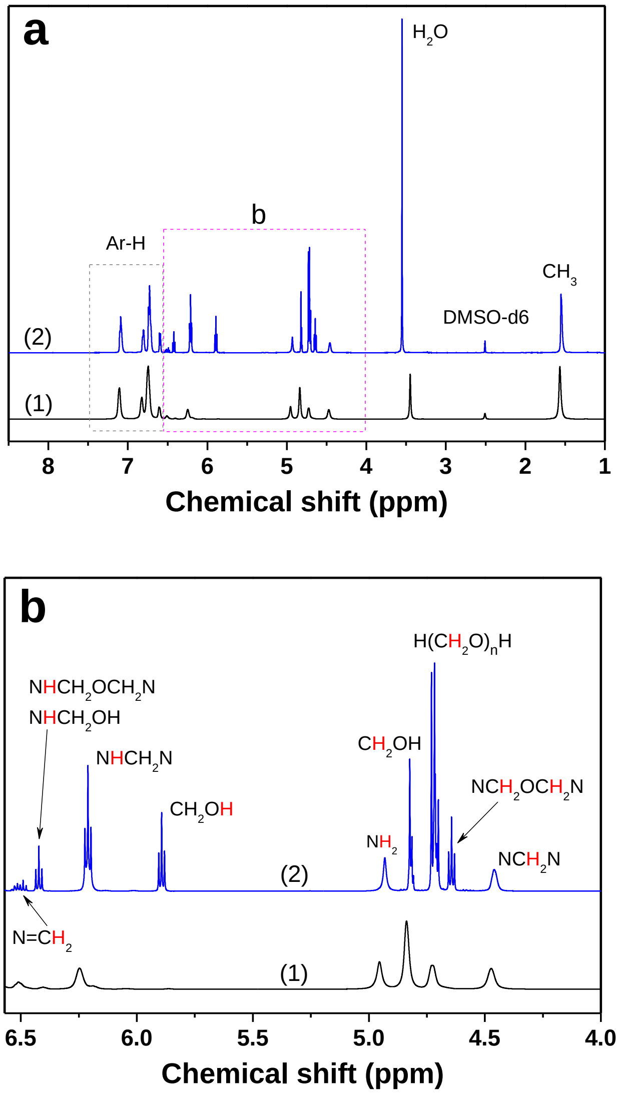

Supplementary Figure 29 | ^1H NMR spectra of BAPP and formaldehyde reacting in (1) anhydrous DMSO-d6 and (2) DMSO-d6 containing 10vol% water. (**b**) is the local amplification of (**a**).

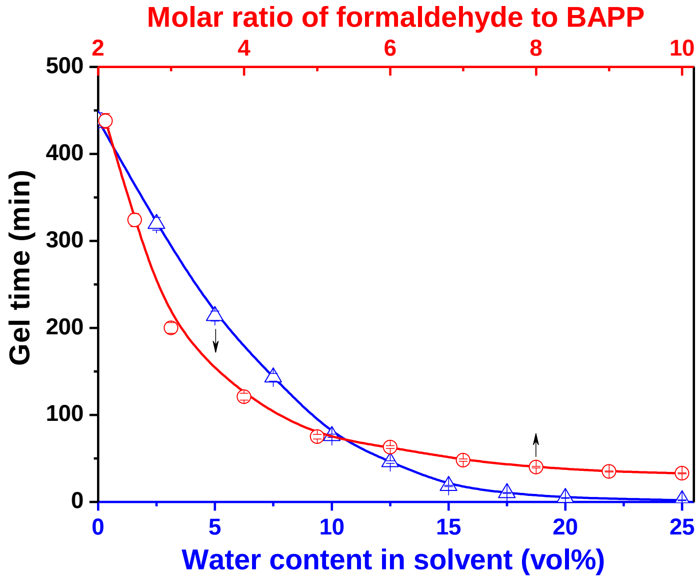

Supplementary Figure 30 | The effects of water content and formaldehyde content on the gel time of BAPP and formaldehyde reaction system. The sample preparation and test procedures are as follows: PFA solution in 10 mL NMP or NMP/H_2O was depolymerized at 80 ^oC for 0.5 h with continuous magnetical stir to prepare the formaldehyde solutionin advance; 8 mmol BAPP was dissolved in 5 mL NMP and introduced into the formaldehyde solution quickly as it was cooled down to 50 ^oC to start the reaction. The mixture was continously stirred until the magnet stopped rotating or white precipitate appeared. Herein, the PFA dosage was fixed at 16.8 mmol to investigate the influence of water, and only NMP as reaction solvent was used to investigate the influence of formaldehyde.

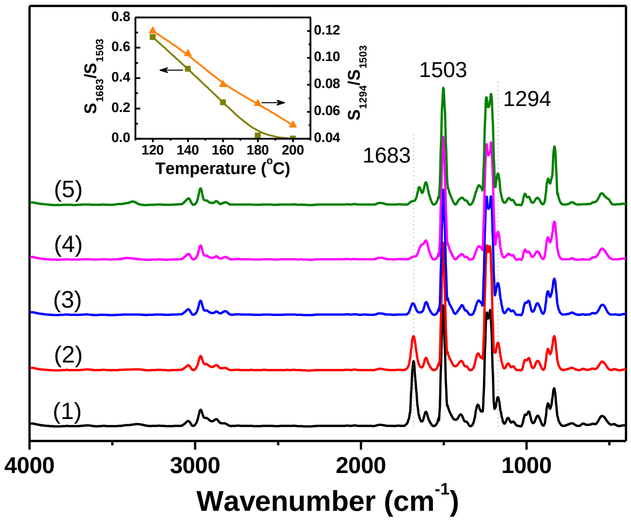

Supplementary Figure 31 | FTIR spectra of the BAPP-PHTs at different times of the post-curing process. The B-stage PHT resin sheets (1) were post-cured by compression molding at different temperatures of (2) 140 °C, (3) 160 °C, (4) 180 °C and (5) 200 °C for 2 h, respectively.

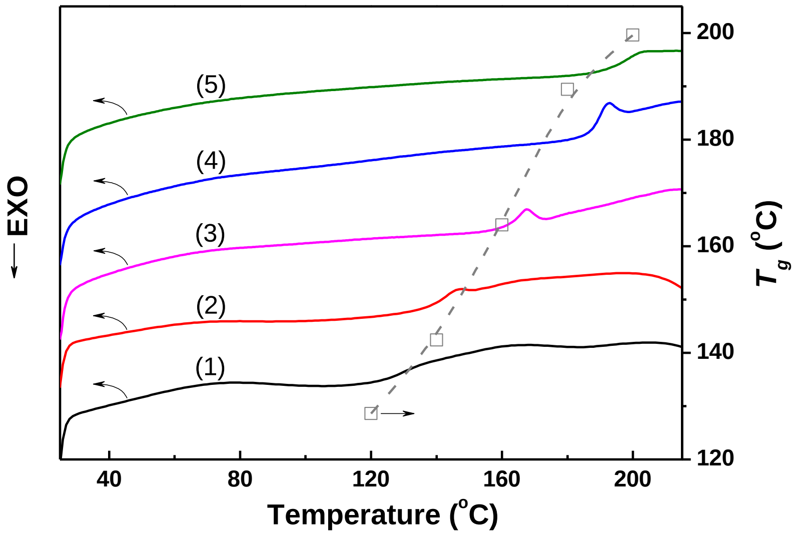

Supplementary Figure 32 | DSC results of the BAPP-PHTs at different times of the post-curing process. The B-stage PHT resin sheets (1) were post-cured by compression molding at different temperatures of (2) 140 °C, (3) 160 °C, (4) 180 °C and (5) 200 °C for 2 h, respectively.

Supplementary Tables

Supplementary Table 1 | Properties of the CF fabrics[a)].

Product designation	Fiber type	Fiber tensile modulus (Gpa)	Fiber tensile strength (GPa)	Fiber elongation (%)	Fiber density (g/cm^3)	Weave	Thickness (mm)	Weight (g/m^2)
CO6142	T300-1000	230	3.53	1.5	1.76	plain	0.15	119
UT-20G	unidirectional high-strength CF	230	3.43	2.1	1.80	unidirectional	0.11	200

[a)] All data are provided by Toray Industries, Inc. (Tokyo, Japan).

Supplementary Table 2 | Characterization results of the mechanical properties of the BAPP-PHT, the unidirectional CF/PHT composite and the cross-ply CF/PHTcomposite.

Samples	The BAPP-PHT	The unidirectional CF/PHT composites	The cross-ply CF/PHT composites
T_g (^oC)	200.1	199.5	198.2
T_d (^oC)	368.5	384.2	376.3
Tensile strength (MPa)	124.7	1806.6	741.2
Young's modulus (GPa)	4.8	141.7	68.3
Elongation at break (%)	3.8	1.4	1.2
Flexural strength (MPa)	151.6	1241.2	829.7
Flexural modulus (GPa)	4	127.4	54.8
Compression strength (MPa)	162.7	343.3	351.5
Short-Beam strength (MPa)		69.1	75.5
Fracture toughness (MPa·$m^{1/2}$)	1.94		

Supplementary Table 3 | The resistance of the BAPP-PHT to various chemical reagents.

Solution	Concentration (wt%)	Change in mass (%)	Change in appearance[a)]	Solution	Concentration (wt%)	Change in mass (%)	Change in appearance
sulfuric acid	98	-100	D	distilled water	100	0	N
sulfuric acid	75	+16.6	E, black	methanol	100	0	N
sulfuric acid	10	0	N	ethanol	100	0	N
sulfuric acid	5	0	N	ethanol	50	0	N
hydrochloric acid	37	-1.2	gray white	acetone	100	0	N
hydrochloric acid	10	0	N	ether	100	0	N
nitrate	70	-100	D	ethyl acetate	100	0	N
nitrate	40	+26.7	E, black	n-heptane	100	0	N
nitrate	10	+6.5	SE, gray	2,2,4-trimethylpentane	100	0	N
acetic acid	99.5	0	N	toluene	100	0	N
acetic acid	5	0	N	xylene	100	0	N
citric acid	10	0	N	NMP	100	0	N
lactic acid	10	0	N	dimethylformamide	100	0	N
sodium hydroxide	40	0	N	dimethyl sulphoxide	100	0	N
sodium hydroxide	1	0	N	tetrahydrofuran	100	0	N
aniline	100	0	N	dichloromethane	100	0	N
ammonium hydroxide	25	0	N	dioctyl Phthalate	100	0	N
ammonium hydroxide	10	0	N	phenol	5	0	N
hydrogen peroxide	30	0	light gray	olive oil	100	0	N
hydrogen peroxide	3	0	N	kerosene	100	0	N
sodium carbonate	20	0	N	gasoline	100	0	N
sodium carbonate	2	0	N	turpentine	100	0	N
sodium chloride	10	0	N	cottonseed oil	100	0	N

[a)] "D": decomposition, "E": expansion, "SE": slight expansion, "N": no change in appearance.

Supplementary Methods

Materials. The plain weave CF cloth (product designation: CO6142) and unidirectional CF cloth (product designation: UT-20G) were purchased from Toray Industries, Inc. (Tokyo, Japan), and the fiber details are listed in Supplementary Table 1.

Characterization Methods. ^{1}H NMR spectra were recorded on a Bruker AVANCE III HD 600 spectrometer (600MHz) using DMSO-*d*6 as a solvent. Fourier transform infrared spectrum (FTIR) was recorded on a Bruker VERTEX70 spectrometer using transmission mode, and the scan times and range were 32 and 400~4000 cm^{-1}, respectively. Dynamic mechanical analysis (DMA) was performed on NETZSCH DMA242C instrument with a heating rate of 5 ^{o}C/min and a frequency of 1 Hz under nitrogen atmosphere. Three-point bending mode was used with sheet dimensions of 55×10×2 mm^3 (L×W×H). Thermo-gravimetric analysis (TGA) was carried out on a NETZSCH TG209F1 apparatus under N_2 atmosphere with a heating rate of 5 ^{o}C/min. A simultaneous thermal analyzer (NETZSCH, STA449C/3/MFC/G) and a FTIR spectrometer (Bruker, TENSOR27) were combined for TG-FTIR analysis and performed at a heating rate of 5 ℃/min from 30 to 600 ℃ under nitrogen. Differential Scanning Calorimetry (DSC) testing was implemented on a NETZSCH DSC204F1 instrument under N_2 atmosphere with a heating rate of 5 ^{o}C/min. X-ray photoelectron spectroscopic (XPS) analysis was fulfilled on a Kratos Axis Ultra-DLD X-ray photoelectron spectroscope in CAE mode with Mono AlK_α radiation of 1486.6 eV. Scanning electron microscopic (SEM) images were acquired on a FEI Quanta 200. Wide-angle X-ray diffraction (XRD) data was collected on a Rigaku MiniFlex X-ray diffractometer operated at 40 kV, 30 mA and CuKα (λ=1.5401 Å) radiation. The liquid wettability of PHT resins was evaluated by static contact angle determined with a contact angle measuring system (OCA20, Dataphysics Instrument) using the sessile-drop method at room temperature. Data were read at 1 min after deposition of 10 μL distilled water or solvent. The final result was calculated as the average of at least five measurements.

The mechanical properties of the BAPP-PHT and the CF/PHT composites were tested using an Instron 5967 and 5984 model materials testing system (USA). According to ASTM D638-14, D790-10 and D695-10 standards, the tensile, flexural and compressive properties of the BAPP-PHT were tested respectively using a loading rate of 5, 5 and 1.3 mm/min at 23±0.5 ^{o}C and 30±5% relative humidity. Twelve specimens were analyzed and the mean

value was adopted for each batch. The thickness and narrow section width of dumbbell shaped specimens for tensile property test are 6 and 3 mm, respectively. The specimen length, width and thickness for flexural property test are 80, 10 and 4 mm, respectively. The flexural span is 64 mm. The specimen square prism side length and height for compressive property test are 12.7 and 25.4 mm, respectively. The fracture toughness of the BAPP-PHT was measured using the compact tension (CT) configuration according to ASTM D5045-14 standard (Supplementary Fig. 1). The CT specimen has a nominal dimension of 37.5×36×8.8 mm^3 (L×W×H). A sharp pre-crack was introduced to each CT specimen by inserting a fresh razor blade at the end of the machined crack and tapping gently with a light hammer. A loading rate of 10 mm/min was adopted and at least twelve specimens were successfully tested for each group.

The tensile property of the CF/PHT composites was tested with a loading rate of 2 mm/min according to ASTM D3039/D3039M-14 standard. For the cross-ply CF/PHT composites, the overall length, width and thickness are 250, 25 and 2.5 mm, respectively. For the 0° unidirectional CF/PHT composites, the overall length, width and thickness are 250, 15 and 1.1 mm, respectively. For the 90° unidirectional composites, the overall length, width and thickness are 175, 25 and 2.0 mm, respectively. The tabs with 50 mm length were firmly bonded at the ends of every specimen with epoxy glue, which were made from the same composites under test. The flexural property of the CF/PHT composites was tested with a crosshead speed of 2 mm/min according to ASTM 790-10 standard. The specimen length, width and thickness are 127, 12.7 and 3 mm, respectively. The span-to-depth ratio is about 32:1. The short-beam strength of the CF/PHT composites was tested with a crosshead speed of 1 mm/min according to ASTM D2344/D2344M-13 standard. The specimen length, width and thickness are 18, 6 and 3 mm, respectively. The span-to-measured thickness ratio is 4. As decribed by Sanchez-Saez et al,[37] the compression tests of the CF/PHT composites were conducted at a loading rate of 0.5 mm/min and the tests were terminated at the complete failure of specimens. The specimen length, width and thickness are 78, 78 and 2 mm, respectively. Tensile test of CF monofilaments was conducted with a crosshead speed of 1 mm/min at room temperature on the basis of ASTM C1557-14 standard. The tested CF bundles and monofilaments were randomly selected from CF cloths and thirty specimens were successfully tested for each group.

Investigation of the polymerization mechanism. The sample preparation and test procedures for anhydrous DMSO-d6 reaction system are: 16 mmol BAPP was dissolved in 10

mL DMSO-d6 in advance; 33.6 mmol PFA and 20 mL DMSO-d6 were added into a flask and continuously magnetically stirred at 80 ^{o}C for 0.5 h, then cooled down to 50 ^{o}C and introuced into the preparative BAPP solution quickly to start the reaction. About 0.3 mL reaction solution was rapidly taken out at certain time interval, immediately diluted with DMSO-d6 and performed NMR analysis. The sample preparation and test procedures for aqueous DMSO-d6 reaction system is the same as the anhydrous system except for introducing 3 mL distilled water and 17 mL DMSO-d6 instead of 20 mL DMSO-d6.

Supplementary Note 1

Characterization data of the BAPP-PHT. The density of the BAPP-PHT is measured as 1.249 g/cm^3 according to ASTM D792-13 standard, which is close to those of epoxy and bismaleimide (BMI) matrix resins. Detailed characterization results of its mechanical properties are shown in Supplementary Figs 2-6 and Supplementary Table 2.

Comparison between BAPP-PHT and the ODA-PHT. By comparison, the BAPP-PHT exhibits lower stiffness (Young's modulus 4.8 GPa) but higher toughness (K_{Ic}=1.94 MPa·m$^{1/2}$) than the PHT resin from ODA (Young's modulus 10.3 Gpa, nonmeasurable K_{Ic})[33]. Besides, T_d of the former (368.5 ^{o}C, Fig. 2e and Supplementary Table 2) is 130.6 ^{o}C higher than that of the latter (synthesized with the same process, 237.9 ^{o}C), which is consistent with the reported result[33]. A comparison between the molecular structures of BAPP and ODA was performed via quantum calculations with the aim to interpret the performance difference of the two PHT resins (Supplementary Fig. 7). Obviously, BAPP possesses double higher molecular weight (410.51 g/mol) than ODA (200.24 g/mol), and moreover it has better structure flexibility. Therefore the density of hexahydrotriazine structure in the BAPP-PHT is less than (about half) that of the ODA-PHT under equal conditions, and BAPP is more conducive to form perfect hexahydrotriazine corsslinked structure than ODA due to the steric hindrance between its two amino groups. The TG-FTIR result shows that the decomposition products of the ODA-PHT at 240 ^{o}C are mainly formaldehyde, which suggests that some weak chemical bonds may exist in this resin, such as $-\overset{|}{N}-CH_2-O-CH_2-\overset{|}{N}-$, and reduce the resin's thermal stability.

Chemical resistance of the BAPP-PHT. The resistances of the BAPP-PHT and the CF/PHT composite to chemical reagents were evaluated according to the ISO 175-2010 and ASTM D543-14 standards, as shown in Supplementary Fig. 8. The specimen with the nominal size of

1 cm×1 cm×3 mm (L×W×H) was loosely bundled with PTFE wire and suspended in the middle of a glass bottle with a small magnet. About 10 mL chemical reagent was added into the bottle and submerged the specimen under slowly magnetic stir. After one week, the remaining sample was taken out (if there were any), washed with water or light naphtha, dried at 50 ^oC for 2 h and weighed. The changes in sample weight and appearance are recorded in Supplementary Table 3. The results show that the BAPP-PHT demonstrates strong resistance to various solvents, oils, alkalis, salt solutions and some strong oxidizing agent solutions. Even 30wt% H_2O_2 oxidizing solution can only turn the sample surface to light gray. But the situation is quite complicated for acids or acid solutions. To be specific, the BAPP-PHT can be completely degraded in strong oxidizing concentrated strong acids, such as concentrated H_2SO_4 solution (about 98wt%) and HNO_3 solution (about 70wt%). Once the concentrated acids were diluted, e.g. 75wt% H_2SO_4 solution, 40wt% and 10wt% HNO_3 solutions, the BAPP-PHT just swells and its weight increases in various degrees, especially for 40wt% and 10wt% HNO_3 solutions, and the higher is the HNO_3 concentration, the more serious are the resin's expansion and oxidation degrees. However, the BAPP-PHT can entirely resist 10wt% and 5wt% dilute H_2SO_4 solutions. This phenomenon is attributed to the stronger oxidation capacity of HNO_3 solution than that of the isocyatic H_2SO_4 solution. For concentrated strong acids without strong oxidizability, such as 37wt% HCl solution, the BAPP-PHT is only slightly degraded (the 7-day mass loss is 1.2wt%) and the surface is slightly corroded and becomes gray white. When the HCl concentration is reduced to 10wt%, the BAPP-PHT shows no change in mass and appearance. For weak acids, the BAPP-PHT is completely free from corrosion.

Overall, the BAPP-PHT has excellent comprehensive mechanical property, thermal stability and chemical resistance. Therefore, it is suitable as the resin matrix candidate for CF reinforced advanced composites.

Supplementary Note 2

Recycling of the BAPP-PHT. In our study, we designed a series of experiments during the recycling process of the BAPP-PHT to investigate the influence of HCl concentration and THF ratio on the first-stage depolymerization time (t_1) of the BAPP-PHT. The analysis results are presented in Supplementary Figs 13-16.

Recycling of the CF/PHT Composites. In our study, two kinds of speciemans individually with the nominal sizes of 1 cm×1 cm×3 mm and 20 cm×20 cm×3 mm (L×W×H) were used to simulate the recovery process of the cross-ply CF/PHT composite. Fig. 4 in the manuscript detailedly reveals the degradation process of the small laminate, and the SEM and XPS characterization results of the CF cloths retrieved at its different depolymerization times are shown elaborately in Fig. 4 and Supplementary Fig. 17, respectively. The device used to study the recycling process of the big laminate and related experiment details are demonstrated in Supplementary Fig. 18, and its whole depolymerization process is visually and detailedly illustrated in Supplementary Fig. 19. Supplementary Figs 20-25 show the comprehensive characterization data of the recycled CF cloths after the matrix resin was completely depolymerized, including the results of SEM, XRD, XPS, CF monofilament tensile test, and photographing analysis.

Supplementary Note 3

Polymerization Mechanism of the BAPP-PHT

As mentioned in the manuscript, the preparation process of the BAPP-PHT is divided into two steps, preparation of partially cured B-stage resin sheets and further compression molding. The hemiaminal dynamic covalent networks (HDCNs) was pre-prepared by the polymerization of aromatic diamines with formaldehyde at low temperatures (~50 ℃)[34]. Then, the transparent B-stage resin sheets were obtained via precuring the prepolymer and removing solvents at 50~120 ℃ on a glass plate. Subsequently, the B-stage resin was heated to 200 ℃ to start a chemical rearrangement and form highly cross-linked PHT covalent networks. The prepolymerization process relates to complex polymerization mechanisms and is found highly dependent on the reaction conditions. There are generally three reported transformation types throughout the entire process: i) amine addition to formaldehyde during the formation of hemiaminal intermediates, ii) water loss from the hemiaminal during the formation of imine intermediates, and iii) amine addition to the imine during the formation of diamine and triamine intermediates.

On this basis, we further study the polymerization kinetics and reaction mechanism of aromatic diamines with formaldehyde via NMR analysis and gel time measurement (Supplementary Figs 26-30). Taken BAPP as an example, the peak area ratios between different reaction groups (H_2O, NH_2 and $NHCH_2N$) and the internal standard methyl groups (CH_3, peak at ~1.55 ppm) corresponds to their relative abundances. As shown in

Supplementary Fig. 27, the abundance of AR-NH_2 (chemical shift at ~4.9 ppm) gradually decreases with the reaction time, and the reaction byproduct water (chemical shift at ~3.5 ppm) shows gradually increasing concentrations accordingly. This phenomenon suggests that the addition of amines occurs simultaneously with the imidization of hemiaminals. The reaction system gels at about 440 minutes and the conversion rate of amine groups reaches 87.7% at this time. If added certain water to the reaction system, e.g. 10vol%, the reaction rate is greatly accelerated and the gel time is reduced to 80 minutes (Supplementary Figs 28 and 30), however, the conversion rate of amine groups before gelling only reaches 85.1%. Just as the anhydrous system, the water concentration also gradually increases with the reaction time in the water-containing reaction system. However, as shown in Supplementary Fig. 30, the higher is the water content, the faster is the reaction rate. This means that the reaction byproduct water can catalyze the subsequent addition reaction between BAPP and formaldehyde, and even demonstrates better catalytic effect than formaldehyde, i.e. this is a self-catalyzed reaction. The reason for this is that all transformations involved C-N formation or C-O bond break concur with the protonation or deprotonation of amine or formaldehyde reactants, which can be facilitated by hydrogen-bonded water molecules[34]. Similar rules are also found when using ODA instead of BAPP. All these results confirm that the reaction between aromatic diamines and formaldehyde under near-neutral conditions pertains to water-promoted self-catalyzed sequential addition condensation, and water can be used to catalyze the polymerization rate, shorten the reaction time, save solvents and avoid excessive use of formaldehyde. Although water catalysis can cause a slight reduction in the conversion rate of amine groups at the gel point, this minor defect can be compensated in subquent post-curing process.

Before gelling, the prepolymer solution was spread onto a glass plate with PTFE frame, and then heated to precure and remove solvents. The colorless transparent B-stage resin sheets were obtained. The sheet thickness can be easily adjusted by changing the prepolymer content in solution and the solution volume spread on the glass plate. Considering the prepolymer gelling process and solvent removal, the optimum sheet thickness is controlled in 0.2~0.6 mm, and in our study the sheet with thickness of 0.3 mm was selected to further produce the BAPP-PHT.

The intermediates occurred during the post-curing process were detected and characterized by recording FTIR spectra and DSC results of the BAPP-PHT post-cured at different temperatures. 3-mm-thick PHT resin specimens were manufactured with 10 layers of resin sheets by compression molding at about 3 MPa and different temperatures. As shown in

Supplementary Fig. 31, using the skeleton stretching vibration peak of benzene at ~1503 cm^{-1} as a reference, the reaction between secondary amine group and hydroxymethyl group of HDCN can be followed by the relative absorption intensity decrease of the deformation vibration peak of hydroxyl groups at ~1294 cm^{-1}. With the cured temperature increasing from 120 to 200 ^{o}C, the relative absorption intensity of the stretching vibration peak of carbonyl group in NMP at ~1683 cm^{-1} gradually weakens and then almost disappears. This indicates that the NMP residual in the B-stage PHT resin can be almost completely removed by heating at 200 ^{o}C for 2 h. The TG-FTIR analysis result shows that the volatile content of the B-stage resin sheets is ~1.8wt%, including NMP and the byproduct water. As shown in Supplementary Fig. 32, with the increase of cured temperature from 120 to 200 ^{o}C, the T_g of the BAPP-PHT increases gradually from 128 ^{o}C to 200 ^{o}C, and further increase of the curing temperature or prolonging the curing time cannot improve T_g significantly. Therefore, the B-stage PHT resin can be nearly completely cured to form highly cross-linked 3D networks by undergoing a chemical rearrangement at 200 ^{o}C for 2 h.

Supplementary References

[1] Gaussian 09, Revision D.01, M. J. Frisch, G. W. Trucks, H. B. Schlegel, G. E. Scuseria, M. A. Robb, J. R. Cheeseman, G. Scalmani, V. Barone, B. Mennucci, G. A. Petersson, H. Nakatsuji, M. Caricato, X. Li, H. P. Hratchian, A. F. Izmaylov, J. Bloino, G. Zheng, J. L. Sonnenberg, M. Hada, M. Ehara, K. Toyota, R. Fukuda, J. Hasegawa, M. Ishida, T. Nakajima, Y. Honda, O. Kitao, H. Nakai, T. Vreven, J. A. Montgomery, Jr., J. E. Peralta, F. Ogliaro, M. Bearpark, J. J. Heyd, E. Brothers, K. N. Kudin, V. N. Staroverov, R. Kobayashi, J. Normand, K. Raghavachari, A. Rendell, J. C. Burant, S. S. Iyengar, J. Tomasi, M. Cossi, N. Rega, J. M. Millam, M. Klene, J. E. Knox, J. B. Cross, V. Bakken, C. Adamo, J. Jaramillo, R. Gomperts, R. E. Stratmann, O. Yazyev, A. J. Austin, R. Cammi, C. Pomelli, J. W. Ochterski, R. L. Martin, K. Morokuma, V. G. Zakrzewski, G. A. Voth, P. Salvador, J. J. Dannenberg, S. Dapprich, A. D. Daniels, Ö. Farkas, J. B. Foresman, J. V. Ortiz, J. Cioslowski, and D. J. Fox, Gaussian, Inc., Wallingford CT, 2009.

[2] GaussView, Version 5.0.9, R. Dennington, T. Keith, J. Millam, Semichem Inc., Shawnee Mission KS, 2009.

[3] Becke, A. D. Density-functional thermochemistry. III. The role of exact exchange. *J. Chem. Phys.* **98**, 5648-5652 (1993).

[4] Lee, C., Yang, W., Parr, R. G. Development of the colle-salvetti correlation-energy formula into a functional of the electron density. *Phys. Rev. B* **37**, 785-789 (1988).
[5] Miehlich, B., Savin, A., Stoll, H., Preuss, H. Results obtained with the correlation energy density functionals of becke and Lee, Yang and Parr. *Chem. Phys. Lett.* **157**, 200-206 (1989).
